# Long-term Contingency Learning Depends on Contingency Awareness

**DOI:** 10.5334/joc.433

**Published:** 2025-02-11

**Authors:** Klaus Rothermund, Lennart Kapinos, Jan De Houwer, James R. Schmidt

**Affiliations:** 1 Friedrich Schiller University Jena, Jena, Germany; 2 Ghent University, Ghent, Belgium; 3 LEAD-CNRS UMR5022, Universitéde Bourgogne, Dijon, France

**Keywords:** Contingency learning, episodic response retrieval, stimulus-response binding, law of recency, contingency awareness, propositional knowledge

## Abstract

We examined long-term contingency learning (CL) in a color classification task with two separate sets of non-overlapping color-word contingencies that were employed in alternating blocks of the task (“alternating blocks paradigm”). Analyzing only the first occurrences of the word distractors in each block provides a pure indicator of long-term CL that is free from recency-based episodic retrieval processes. A high-powered (n = 110), pre-registered study revealed evidence for reliable long-term color-word CL. This long-term CL effect depended on contingency awareness, indicating that genuine long-term CL is influenced by propositional knowledge.

Color-word contingency learning (CL; Schmidt et al., 2007) is an established paradigm of human associative learning (for a recent review, see Schmidt, 2021). In this task, word stimuli have to be classified according to the color in which they are printed, with each word appearing mostly in one specific color and only rarely in other colors. Although the words are irrelevant for the task, high-frequency color-word combinations are classified faster than low-frequency combinations.

Early accounts of CL proposed that the effect is based on an implicit learning of abstract S-R contingencies, reflecting an automatic form of behavioral control (Schmidt et al., 2007). Subsequent findings gathered with this paradigm, however, suggest that CL effects can be explained to a large degree in terms of a recency-based retrieval of episodic stimulus-response bindings (Giesen et al., 2020; Güldenpenning et al., 2024; Jiménez et al., 2022; Rothermund et al., 2022; Rudolph et al., 2024; Rudolph & Rothermund, 2024; Schmidt, Giesen, et al., 2020; Xu & Mordkoff, 2020). This instance-based retrieval mechanism explains CL effects without assuming that contingencies proper influence behavior. Instead, the presence of contingencies leads to a biased retrieval of individual episodes in which the more frequent word-color combinations had been presented. This biased retrieval produces facilitation for trials with frequent word-color combinations due to a more likely retrieval of a matching response. Similarly, retrieving a response from a previous high frequency episode will interfere with responding on low frequency trials. What looks like an effect of a learned regularity at first sight can thus actually be explained by a simple retrieval of the most recent episode in which the current word stimulus occurred (the “law of recency”; Giesen et al., 2020; Schmidt, Giesen, et al., 2020).

Such an explanation of CL in terms of recency-based episodic retrieval conflicts with the claim that CL reflects learning proper. The CL effect would not fall under a definition of learning according to which learning consists in behavior adapting to environmental regularities (but see De Houwer & Hughes, 2020). A CL effect that reflects the influence of only a single, most recent episode by definition does not constitute a contingency because contingencies are defined in terms of relative frequencies or probabilities that have no meaning when applied to a single event. Recency-based episodic retrieval also has no lasting effects on behavior, since encountering the next episode could completely reverse the CL effect, leaving no enduring traces of learning.

More recent evidence, however, suggests that episodic retrieval processes do not explain the entire CL effect, and that a small but reliable CL effect remains after statistically controlling for episodic retrieval processes (Rudolph et al., 2024; Rudolph & Rothermund, 2024; see also Jiménez et al., 2022; Xu & Mordkoff, 2020). Such a residual CL effect goes beyond effects of a single event and thus is a candidate for the influence of actual contingencies (across multiple events) on behavior. Statistically controlling for episodic retrieval effects, however, has some limitations. Specifically, the predictor that codes retrieval in the regression equation is only an imperfect estimator of the direction and strength of the actual retrieval processes. For instance, when coding the influence of retrieval it is typically assumed that (a) episodic retrieval influences responding to the same degree in each trial, (b) facilitation and interference effects are of equal magnitude, (c) only the last matching episode determines retrieval, and (d) only episodes with an identical (but not with a similar) stimulus are retrieved. These assumptions are necessary in order to come up with a tractable operationalization of retrieval, which cannot be observed or manipulated directly. These simplifying assumptions, however, often do not fully apply in real situations (e.g., retrieval is a stochastic process that may or may not operate in a specific trial, it might bypass the last occurrence, or it may retrieve an episode based on a mere similarity or feature overlap with the current situation). Making these strong assumptions when applying the statistical approach thus introduces error variance into the predictor variable that codes retrieval. The variable that codes retrieval in the regression equation is only an imperfect indicator of actual retrieval, which will lead to an underestimation of the actual strength of the retrieval effect. By implication, *under*estimating the retrieval effect will lead to an *over*estimation of the strength of the residual CL effect, which has been assumed to reflect genuine CL (for a similar reasoning, see Klauer et al., 1998; Schmidt et al., 2014). It could thus be possible that the residual CL effect after statistically controlling for the influence of episodic retrieval results from an incomplete controlling for influences of actual episodic retrieval. The residual effect might still reflect the operation of episodic retrieval processes that have not been properly captured by the predictor variable in the regression, rather than indicating genuine CL.

The present study thus takes another route to assess genuine CL effects that are free from episodic retrieval processes. Rather than statistically – that is, imperfectly – controlling for episodic retrieval, we focused on long-term CL effects. Specifically, we investigated CL effects across a temporal distance that by far exceeds the typical duration on which automatic episodic S-R retrieval processes operate. We additionally controlled for any remaining effects of episodic retrieval by perfectly balancing the response relation of the long distance trials to the last occurrences (for procedural details, see below). The resulting long-term CL effects thus provide us with an indicator of genuine CL effects that are free from recency-based episodic retrieval. If we still find a CL effect for these long distance trials, across temporal intervals that clearly exceed the time frame of recency-based episodic retrieval processes, then this would be strong evidence for genuine CL that is independent of recency-based episodic retrieval. If, however, CL effects should disappear after a longer temporal interval in which a stimulus was not encountered, leaving no lasting traces of learning, then this would speak in favor of the hypothesis that CL effects are entirely due to recency-based episodic retrieval, and thus do not reflect learning proper.

A somewhat related approach was taken in a study by Schmidt, De Houwer, et al. (2020) who investigated whether effects of initially acquired contingencies survived after removing or changing the contingencies. Their findings suggest that – at least for highly overlearned initial contingencies – reliable CL effects can be found even in a novel context in which the original contingency no longer holds, ruling out an explanation of the CL effect in terms of recency-based retrieval.

Although the research of Schmidt, De Houwer, et al. (2020) provided evidence for genuine CL effects, it did not address the question of the mechanisms that underlie these effects. Other research, based on the approach of statistically controlling for effects of recency-based retrieval, revealed that residual CL effects were dependent on contingency awareness (Rudolph et al., 2024; Rudolph & Rothermund, 2024). This was taken to indicate that genuine CL is based on abstract, de-contextualized propositional knowledge about the word-color contingencies (De Houwer, 2009, 2014; Mitchell et al., 2009). The fact that no residual CL effect was found for S-R contingencies of which individuals were unaware speaks against an influence of association formation^1^ as a source of CL. Association formation, that is, the emergence of excitatory S-R links in memory, is typically assumed to develop gradually and asymptotically, based on the mere frequency of experiencing a specific S-R combination (e.g., Rescorla, 1972). The mere frequencies of co-occurrence, however, were identical for S-R pairings with and without contingency awareness, ruling out an explanation of the awareness-dependent CL effect in terms of association formation. Investigating the role of contingency awareness for long-term CL effects would provide us with another independent test of the nature of the representations underlying genuine CL.

## The present study

**Figure 1:**
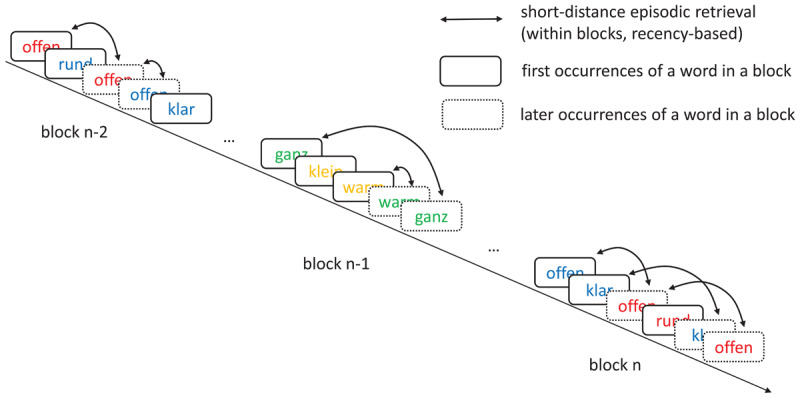
The alternating blocks paradigm: Two non-overlapping sets of color-word contingencies are presented in alternating blocks of the experiment. Responding to the first occurrences of a word within each block constitutes a pure measure of long-term contingency learning. Responding to later occurrences of a word within a block reflects a mixture of long-term learning and short-term retrieval.

To assess pure long-term CL effects that are free from recency-based episodic retrieval, we developed a new variant of the color-word CL task, the so-called “alternating blocks paradigm”. In this version of the paradigm, two non-overlapping sets of color-word contingencies are presented in alternating blocks of the task (Figure 1). To compute a pure indicator of long-term contingency learning that is free from short-term retrieval processes, CL effects (i.e., the RT difference between high- and low-frequency color-word combinations) were computed only on the basis of the very first occurrences of the word distractors in each block. This effect is not influenced by recency-based retrieval, since there are no matching episodes that occurred immediately before the current trial within the same block. Since the immediately preceding block consisted of a different set of words and colors, the last matching episode for these trials thus occurred very long ago, in our case more than 30 trials before the current trial (approx. 1–2 minutes), a distance which has previously been shown to completely eliminate effects of the last occurrence of the stimulus on current performance (Giesen et al., 2020; see also other studies showing that recency-based retrieval is limited to short time intervals, with effects vanishing after intervals ranging between two and ten seconds; Frings, 2011; Moeller & Frings, 2017; 2021; Moeller et al., 2016).^2^ In addition, we also controlled for the last occurrences of the words presented for the first time in a block in the n-2^nd^ blocks, making sure that same and different response episodes were equally likely for both high and low frequency word/color combinations, which eliminates any possible confound between contingencies and episodic response retrieval for words that appear for the first time in a block. Each participant went through multiple repetitions of the two sets of blocks, which resulted in a reliable measure of long-term learning, although only four trials per block entered into the indicator of long-term learning (i.e., the first occurrences of the four words that made up a set of words for the contingency rules within a block).

In addition to providing a pure indicator of genuine long-term CL, the paradigm also allows us to compute CL effects for the later occurrences of words within each block. These trials can be used to compute residual CL effects, according to the standard statistical approach, after controlling for the influence of recency-based retrieval. Conducting these analyses allows us to compare results for pure long-term CL with results of residual CL effects (controlling for recency-based retrieval) within the same experiment. Analyses for residual CL effects are structurally similar to analyses that have been reported in previous articles of residual CL effects and allow us to compare our findings with previous studies that employed the statistical approach to obtain CL effects that are free from recency-based episodic retrieval (Giesen et al., 2020; Schmidt, Giesen, et al., 2020; see also Güldenpenning et al., 2024; Rudolph et al., 2024; Rudolph & Rothermund, 2024; Xu & Mordkoff, 2020).

To investigate the underlying sources of genuine long-term CL, as well as residual CL effects, we assessed contingency awareness at the end of our study separately for each word stimulus. This allowed us to investigate whether long-term CL effects and residual CL effects were modulated by knowledge of the contingencies.

## Method

### Ethics approval, pre-registration, and open access

Ethical approval was granted by the Ethics Committee of the FSU Jena (FSV 20/005). Prior to data collection, the exact method, design, hypotheses, data preparation, and planned analyses were pre-registered online (https://aspredicted.org/tr326.pdf). All data and analyses scripts are available at https://osf.io/q6eur/?view_only=cbb5fd11f29742e29944245e3bfa3fcd.

### Required sample size and a-priori power calculations

Based on a recommendation by Brysbaert (2019), we aimed at collecting data from a sample of n = 110 participants, which allowed us to detect a difference in simple effects (d = .4 vs. d = .0) in a 2 × 2 interaction (high vs. low frequency trials × same vs. different response during the last occurrence of the word distractor) with a power of .8.

#### Participants

110 participants were recruited online from Prolific Academic (53 female, 52 male, 5 other; *M_age_* = 22.9 years). All participants were pre-screened to be native German speakers, aged between 18 and 30 years, using Windows as an operating system and running the experiment on a notebook or desktop computer. The experiment had an average duration of slightly less than 25 minutes and participants were compensated with £3.75 for taking part. All participants gave informed consent prior to taking part in the study.

#### Design

The experimental design for testing long-term CL effects for the first occurrences of words within blocks consisted of a single repeated measures factor (contingency: high vs. low frequency combination of color and word in the current trial). The experiment also allowed us to test residual CL effects within blocks with a 2 (contingency: high vs. low frequency combination of color and word in the current trial) × 2 (last occurrence: same vs. different response during the last trial in which the word had been presented) repeated measures design, with all factors being manipulated within participants. For both analyses, contingency awareness (CA: correctly vs. incorrectly identified word-color relation), as measured at the end of the experiment, was included as an additional factor to test whether CL effects depended on CA. Reaction times (RT) served as the dependent variable of interest. In addition, contingency awareness was assessed for each word at the end of the experiment, which allowed us to test whether CL effects depended on contingency awareness (CA).

#### Materials and procedure

The experiment was programmed with E-Prime 3 and was converted for online data collection with E-Prime Go 1.0. At the start of each experiment, demographic information (gender, age, handedness) was collected, followed by the consent page. If participants consented to take part, instructions followed; otherwise, the study was terminated.

Participants’ task was to categorize the color of letter strings by pressing one of two keys (‘D’ or ‘L’) on a keyboard. They were informed that they would perform two different binary color classification tasks in alternating blocks (red vs. yellow, blue vs. green). The same two keys were used for both color pairs. The labels of the two colors that defined the color classification task for the respective block were always shown in the top left and top right corner of the screen throughout the entire block to indicate which of the two colors was assigned to the left and right key, respectively.

The stimuli for the color classification task were eight neutral German adjectives with a length of 4 to 6 letters that were divided into two sets of four words (set 1: ‘offen’ [open], ‘weich’ [soft], ‘klar’ [clear], ‘rund’ [round]; set 2: ‘warm’ [warm], ‘leicht’ [light], ‘ganz’ [whole], ‘klein’ [small]). The two sets of words were presented in alternating blocks of the task, with one set of words always being presented in red vs. yellow color in the odd-numbered blocks, while the other set was always presented in blue or green color in the even-numbered blocks. All stimuli were presented in the center of the screen in 18 pt. Times New Roman font against a black background. Each block started with two trials in which a string of four Xs was presented once in each of the two colors of the current color categorization task to familiarize participants with the color/key assignments of the respective block.

Each participant received 22 blocks of 26 trials each (24 word trials plus two starting trials with XXXX strings instead of words). The first two blocks were considered as practice blocks that were used to establish the color-word contingencies. The experiment was programmed to terminate after the practice blocks if accuracy was below 80 percent, which did not happen. The 24 trials of each block in which a word was presented were made up of six presentations of each of the four words of the respective word set (see Table 1 for an overview of the contingency manipulations and block structure). The sequence in which the 24 words were presented was randomized for each block. To establish color-word contingencies, each word was presented three times in one color (high frequency combination) and only once in the other color (low frequency combination) of the respective block. Each of the two colors of a pair was assigned to two high-frequency words and to two low frequency words, and each of the four words had one high- and one low-frequency color. For each participant, the same contingencies between words and colors were realized throughout the entire experiment. Across participants, the assignment of colors to high- vs. low-frequency words was counterbalanced. To ensure complete independence of long term CL and episodic retrieval of the response of the last episode, the first and the last presentations of the four words of a set within each block were always balanced with regard to their color-word assignment, with two words appearing in their high-, and the other two words appearing their low-frequency colors, yielding an equal amount of trials in which the first occurrences retrieved the same or a different response from the last occurrence of the word in the n-2^nd^ block, for both high and low frequency first occurrences. In total, these measures resulted in an overall contingency of 2:1. That is, each word had a 3:1 contingency with regard to color assignment for the four central (2^nd^ to 5^th^) presentations of the word within each block (in terms of differences in conditional probabilities [e.g., Allan, 1980], the contingency for a word predicting the occurrence of its associated color compared to the base rate for the central presentations would be ΔP = 60/80–100/240 = .75 – .42 = .33), but had a 1:1 contingency when considering only the first and the last (i.e., 6^th^) appearance of the word in a block (i.e., a contingency of zero, ΔP = 10/20–30/60 = .5 – .5 = 0]). In sum, this resulted in an overall contingency of 4:2 (i.e., 2:1) between words and colors (ΔP = 80/120–160/360 = .67 – .44 = .23).

**Table 1:** Color-word contingency manipulations in the color classification task. Numbers indicate frequencies of occurrence for color-word combinations for different parts of each block (initial part of a block [1*^st^* occurrences], central part [2*^nd^*–5^th^ occurrences], and final part of a block [6^th^ occurrence]) across the 20 blocks of the task; numbers in parentheses indicate the frequency of occurrences within a single example block.


COLOR	WORD SET 1				WORD SET 2			
	
	OFFEN	WEICH	KLAR	RUND	WARM	LEICHT	GANZ	KLEIN

1^st^ occurrences								

red	10 (1)	10 (0)	10 (1)	10 (0)	/	/	/	/

yellow	10 (0)	10 (1)	10 (0)	10 (1)	/	/	/	/

blue	/	/	/	/	10 (1)	10 (0)	10 (1)	10 (0)

green	/	/	/	/	10 (0)	10 (1)	10 (0)	10 (1)

2^nd^–5^th^ occurrences								

red	60 (3)	60 (3)	20 (1)	20 (1)	/	/	/	/

yellow	20 (1)	20 (1)	60 (3)	60 (3)	/	/	/	/

blue	/	/	/	/	60 (3)	60 (3)	20 (1)	20 (1)

green	/	/	/	/	20 (1)	20 (1)	60 (3)	60 (3)

6^th^ occurrences								

red	10 (0)	10 (1)	10 (0)	10 (1)	/	/	/	/

yellow	10 (1)	10 (0)	10 (1)	10 (0)	/	/	/	/

blue	/	/	/	/	10 (0)	10 (1)	10 (0)	10 (1)

green	/	/	/	/	10 (1)	10 (0)	10 (1)	10 (0)

Overall								

red	80 (hc)	80 (hc)	40 (lc)	40 (lc)	/	/	/	/

yellow	40 (lc)	40 (lc)	80 (hc)	80 (hc)	/	/	/	/

blue	/	/	/	/	80 (hc)	80 (hc)	40 (lc)	40 (lc)

green	/	/	/	/	40 (lc)	40 (lc)	80 (hc)	80 (hc)


*Note*. hc, high contingency color-word combinations; lc, low contingency color-word combinations. The specific assignment of words to colors represents only one instance of the counterbalanced design.

Each trial of the color classification task started with a fixation cross (500 ms), followed by a word (or the letter string XXXX) presented in white color for 500 ms, after which it changed its color into one of the two colors of the color categorization task. The words appeared in white before being colored in order to give the retrieval process a head start before the color information could determine the response, which makes the task more sensitive in detecting an influence of retrieval processes (see, e.g., Giesen et al., 2020; Schmidt & De Houwer, 2016). The stimulus then remained on screen until a color response was made. Inaccurate responses elicited a feedback message (“*Fehler!*” [Error] followed after 1 second by “*Weiter mit ‘Leertaste’*” [Continue by pressing the space bar]). Feedback was displayed in white font. Then, the next trial started. After the 5^th^, 10^th^, and 15^th^ block of the task, participants were asked to take a short break, the duration of which they could determine for themselves, and to continue working on the task by pressing the space bar.

After completing all blocks of the color classification task, contingency awareness was assessed by presenting each word in each of the four colors on the screen. For each word, participants had to indicate in which color the respective word had been most often presented during the experiment by clicking on the respective word/color combination. Chance responding on the awareness test would result in 25% correct responses (with only one out of four color options being correct). On average, participants identified the correct color for 42.4% of the trials, which is above chance, *t*(109) = 8.31, *p* < .001, but is far from being perfect.

### Data preparation and analysis plan

Prior to analyses, trials in which a color classification error occurred in the current trial (3.1%) or during the most recent trial in which the word of the current trial had occurred (4.6%) were discarded. Also, responses faster than 200 ms or slower than 1.5 interquartile ranges above the 75^th^ percentile of the individual RT distribution were regarded as RT outliers (Tukey, 1977) and were also excluded (3.2%).

After errors and outlier trials had been eliminated, data were analyzed with hierarchical multi-level regression analyses, treating trials as nested within subjects, while allowing for random intercepts to control for differences in response speed between participants. RT was the dependent variable of interest. Each trial was coded according to whether (a) the current word/color combination reflected a high (frequent) vs. low (infrequent) contingency combination (CL: hc vs. lc), (b) whether the same or a different response had been given for the word of the current trial during its most recent occurrence (ER: same vs. different), and (c) whether the word/color contingency had been correctly identified for the respective word of the current trial during the contingency awareness assessment at the end of the experiment (CA: aware vs. unaware). All predictors indicated a contrast between two conditions and were coded to have (1) a mean of zero across all trials within the analysis, and (2) a difference of 1 between the two weights (see Rudolph & Rothermund, 2024).^3^ Thus, the resulting regression coefficients reflect the difference between the two conditions (in milliseconds), and main effects and interactions of the predictors can be interpreted simultaneously due to the fact that the centered predictors and their products (interactions) are orthogonal. The predictor variables and their interactions were entered in a stepwise fashion into the regression equation to see how introducing additional predictors (e.g., episodic retrieval) might change the effect of the CL effect. Separate multilevel regression analyses were conducted for the first and for the later occurrences of the words within each block to test for long-term CL (across blocks) and standard CL effects (within blocks).

## Results

### Long-term color-word contingency learning

In a first analysis, we tested whether there were effects of pure long-term CL, in which only trials with first occurrences of a word in each block were entered (see Table 2). In a first step, CL was entered as a predictor in the analysis, which yields a significant long-term CL effect: On average, participants responded 5 ms faster in high contingency compared to low contingency trials, *t*(7966) = –2.88, *p* < .01. In a second step, contingency awareness (CA) and its interaction with CL were entered as additional predictors into the model. The interaction of contingency awareness with long-term CL yielded a significant effect, *t*(7974) = –2.61, *p* < .01, indicating that the long-term CL is modulated by contingency awareness.

**Table 2:** Results of a stepwise multi-level regression analysis predicting RT based on contingencies (CL, step 1), contingency awareness (CA) and its interaction with CL (step 2).


PREDICTOR	MODEL 1	MODEL 2

Intercept	403*** [393.5, 411.5]	403*** [393.6, 411.6]

CL (hc vs. lc)	–5** [–7.8, –1.5]	–5** [–7.7, –1.4]

CA (correct vs. incorrect)		–4* [–7.3, –0.2]

CL × CA		–9** [–14.9, –2.1]

BIC	92425	92407

∆ BIC	–	–18


*Note*. *p < 0.05, **p < 0.01, ***p < 0.001. CL, Contingency learning; hc/lc: high/low contingency trials. CA, contingency awareness; correct/incorrect: identification of the typical word/color combination. BIC, Bayesian information criterion. We implemented a person specific intercept to control for individual differences in RTs. All other variables were implemented on a trial level. Values in brackets indicate the 95% confidence interval (lower and upper limit) for each regression weight. Regression weights (*ß*) reflect the difference in milliseconds between the conditions that define a contrast.

To follow up on the CL × CA interaction, we analyzed the strength of the long-term CL effect separately for each awareness level. For trials with words for which the color that was linked to the word was correctly identified, a significant long-term CL effect emerged (*M* = –10 ms, *t*[3364] = –4.05, *p* < .001), whereas no long-term CL was found for those trials in which words were presented for which the respective participant was unable to recognize the word-color contingency (*M* = –1 ms, *t*[4521] < 1).

### Standard color-word contingencies (within blocks)

In a second analysis, we tested standard and residual CL effects, using the statistical approach to control for effects of episodic retrieval. All later occurrences of a word that had a predecessor within the same block (2^nd^ to 6^th^ occurrences of each word within a block) were entered into the analysis (see Table 3). In a first step, CL was entered as a predictor in the analysis, which yields a robust CL effect: On average, participants responded 8 ms faster in high contingency compared to low contingency trials, *t*(39867) = –10.32, *p* < .001. In a second step, episodic retrieval (ER, same vs. different response during the last occurrence of the word) was entered as an additional predictor. Entering ER produced a robust effect for recency-based retrieval with responses being 13 ms faster for trials that required the same response as during the last occurrence compared to trials in which a different response had to be given as during the last appearance of the word, *t*(39869) = –16.86, *p* < .001. Entering ER rendered the residual CL effect insignificant (*M* = –2 ms, *t*[39868] = –1.85, *p* = .06). In a final third step, contingency awareness (CA) and its interactions with CL and ER were entered as additional predictors into the model. The CL × CA interaction was significant, *t*(39872) = –3.96, *p* < .001, indicating that the residual CL effect was modulated by contingency awareness.

**Table 3:** Results of a stepwise multi-level regression analysis predicting RT based on contingencies (CL, step 1), episodic retrieval (ER, step 2), contingency awareness (CA) and its interaction with CL (step 3).


PREDICTOR	MODEL 1	MODEL 2	MODEL 3

Intercept	393*** [385.3, 401.6]	393*** [385.3, 401.6]	393*** [385.3, 401.6]

CL (hc vs. lc)	–8*** [–9.2, –6.3]	–2 [–3.2, +0.1]	–2 [–3.2, +0.1]

ER (matching vs mismatching)		–13*** [–14.3, –11.3]	–13*** [–14.3, –11.3]

CA (correct vs. incorrect)			–0 [–1.8, +1.2]

CL × CA			–5*** [–8.4, –2.5]

BIC	451971	451687	451670

∆ BIC	–	–284	–17


*Note*. *p < 0.05, **p < 0.01, ***p < 0.001. CL, Contingency learning; hc/lc: high/low contingency trials. ER, episodic retrieval; matching/mismatching response during the last occurrence of the word; CA, contingency awareness; correct/incorrect: identification of the typical word/color combination. BIC, Bayesian information criterion. We implemented a person specific intercept to control for individual differences in RTs. All other variables were implemented on a trial level. Values in brackets indicate the 95% confidence interval (lower and upper limit) for each regression weight. Regression weights (*ß*) reflect the difference in milliseconds between the conditions that define a contrast.

Following up on the CL × CA interaction, we analyzed the strength of the residual CL effect separately for each awareness condition. For trials with words for which the color that was linked to the word was correctly identified, a significant residual CL effect emerged (*M* = –5 ms, *t*[16950] = –4.43, *p* < .001), whereas no residual CL was found for those trials in which words were presented for which the respective participant was unable to recognize the word-color contingency (*M* = +1 ms, *t*[22818] = 1.16, *p* = .25).

## Discussion

In the present study, our primary goal was to investigate long-term learning of stimulus-response contingencies that is free from the influence of episodic retrieval. For this purpose, we developed a variant of the color-word contingency learning paradigm (Schmidt et al., 2007), in which two non-overlapping sets of color-word contingencies are presented in multiple alternating blocks of the experiment (the alternating blocks paradigm). Focusing only on the first occurrences of the word stimuli in each block provides us with an indicator of CL that is free from the influence of previous matching episodes that occurred very recently. A first major finding of our study is that we did find evidence for pure long-term CL: Reliable effects of CL were obtained even in a situation where the current word stimulus had not been encountered for a longer time interval during which more than 30 trials comprising different words and different colors had been presented. Our findings replicate and extend previous findings by Schmidt, De Houwer, et al. (2020) who also found reliable CL effects after removing or changing initial contingencies, and which thus cannot be explained by a retrieval of recent episodes.

Having established long-term CL as a reliable phenomenon, another question we wanted to pursue in our study was to test hypotheses regarding the underlying processes that mediate this type of learning. For this purpose, we tested a set of opposing predictions that were derived from either an association formation account, or from an account of CL in terms of abstract propositional knowledge (De Houwer, 2014; Mitchell et al., 2009). The crucial finding in this regard is that the long-term CL effect was modulated by contingency awareness: A significant long-term CL effect was obtained for trials comprising words for which the respective word/color contingency could be correctly identified, whereas no long-term was visible for trials comprising words for which the contingency could not be identified correctly. This pattern supports an explanation of long-term CL in terms of propositional knowledge (e.g., De Houwer, 2009, 2014; Mitchell et al., 2009). An association formation account that explains long-term learning in terms of a gradual, automatic emergence of excitatory links, based on the frequency of exposure to S-R pairings (e.g., Rescorla, 1972), has difficulties explaining these findings since the actual frequency of exposure to S-R pairings was identical for all words. Such an association formation account of long-term CL would thus have predicted a significant CL effect that is independent of awareness, or that is present also for trials without contingency awareness.

The pattern of findings for long-term CL effects was nicely paralleled by the analysis of residual CL effects for the later, repeated occurrences of words within blocks. Replicating results from previous studies, a substantial portion of the standard CL effect was explained by short-term episodic retrieval effects (Giesen et al., 2020; Güldenpenning et al., 2024; Rudolph et al., 2024; Rudolph & Rothermund, 2024; Schmidt, Giesen, et al., 2020; Xu & Mordkoff, 2020). The residual CL effect – after controlling for episodic retrieval – just failed to become significant. However, again replicating recent studies (Rudolph et al., 2024; Rudolph & Rothermund, 2024), the residual CL effect was modulated by contingency awareness. Similar to the pattern that was obtained for long-term CL, we found a significant residual CL effect for trials comprising words for which the word/color contingency was identified correctly, whereas there was no residual CL effect for trials without contingency awareness. Thus, although the main effect of CL was not significant in the analysis of residual CL effects^4^, the core result of a modulation of genuine CL effects by contingency awareness was obtained both for pure long-term CL effects (first occurrences of words within blocks) as well as for the residual CL effects (later, repeated occurrences of words within blocks).

In sum, independently of whether we investigated long-term or residual CL effects, our study provides evidence for reliable genuine CL effects, the existence of which depended on contingency awareness. These findings support and extend recent findings that also support an account of genuine S-R CL in terms of propositional knowledge (Rudolph et al., 2024; Rudolph & Rothermund, 2024).

### Limitations

Our study provided evidence for long-term CL, and its dependence on contingency awareness. This does not mean, however, that our results allow for the conclusion that contingency awareness is necessary for long-term CL. Such a conclusion relies on a non-significant long-term CL for words for which the word-color contingency was not identified correctly. Given that the long-term CL effect was comparatively small in absolute terms, not detecting such an effect in the absence of contingency awareness might be a power problem. In addition, it has to be noted that the overall contingency that was implemented in our study (2:1; ΔP = .23) was not very strong either. It thus cannot be ruled out that an effect of long-term CL even in the absence of contingency awareness might be obtained for stronger contingency manipulations. Alternatively, it could also be that stronger contingencies may produce stronger long-term CL effects, based solely on increased levels of contingency awareness. Future research is definitely needed to more systematically address whether contingency awareness is necessary for long-term CL.

Another obvious limitation of our studies regards the limited time range that was used to study long-term CL. “Long” in our study meant an interval of roughly one minute. Although this can be considered as a very long interval compared to the intervals that are the default in experiments investigating CL effects and episodic stimulus-response retrieval (e.g., Frings et al., 2020; 2024), it is by no means long when considering typical learning processes in everyday life (e.g., skill acquisition, learning a language, developing a habit), where different learning episodes are typically separated by hours, days, or even weeks. In order to more confidently transfer our results to these forms of learning, studies employing much longer time intervals have to be conducted.

Another boundary condition that limits the range of conclusions we can draw from our study is that we only analyzed learning effects with regard to response speed in a simple binary classification task. Obviously, we cannot yet draw any inferences on long-term learning that can be obtained with, for example, free choice or judgment tasks.

## Conclusion

Explaining the stability and persistence of behavior requires an understanding of long-term learning. Our study demonstrates genuine long-term CL that is completely free from the influence of retrieval processes targeting recent episodes. Importantly, our findings support an explanation of these genuine long-term learning effects in terms of propositional knowledge. Sustainable learning effects did not emerge automatically as a result of mere exposure to environmental contingencies but depended on the conscious awareness of these contingencies regarding the co-occurrence of words and colors.

## Data Accessibility Statement

All data and analyses scripts are available at https://osf.io/q6eur/?view_only=cbb5fd11f29742e29944245e3bfa3fcd.

## Footnotes

We use the term “association formation” in a narrow sense, in which associations denote excitatory links between mental representations of stimuli and/or responses. Association formation in this narrow sense should not be equated with “associative learning”, which is a broad descriptive label denoting any form of learning reflecting adaptive changes in an organism’s behavior in response to environmental contingencies (e.g., De Houwer & Hughes, 2020; Shanks, 1995), regardless of the mediating mechanism that can explain such changes.

Our paradigm still allows us to systematically check for influences of the last matching episode on performance across blocks, that is, for trials which occurred more than 30 trials ago. As expected, however, we did not find any evidence for these long-term retrieval effects. On average, responses were even 1 ms *slower* for trials in which the last response that had been given to the word in the n-2^nd^ block was repeated in the current trial, but this difference was clearly not significant, *t* < 1.

The general formulas that will satisfy these standards are: \[
{{w}_{1}}\ = \ \frac{{{f}_{2}}}{\left({{f}_{1}} \ + \ {{f}_{2}}\right)}\], and (2) \[
{{w}_{2}} \ = \ \frac{-{{f}_{2}}}{\left({{f}_{1}} \ + \ {{f}_{2}}\right)}\] where *w_1_* and *w*_2_ are the regression weights that define a contrast, and *f_1_* and *f*_2_ are the number of trials per condition.

We can only speculate why the absolute size of the overall residual CL effect is smaller than the absolute size of the overall genuine long-term CL effect. One possible reason might be that the design of our study was balanced for long-term CL, with an equal number of trials entering into the high and low frequency condition, whereas for the within-blocks analyses (residual CL effect), the design was imbalanced, with high frequency trials outnumbering the low frequency trials by the factor 3.

## Author Information

Klaus Rothermund and Lennart Kapinos, Friedrich-Schiller-Universität Jena, Jena, Germany; Jan De Houwer, Ghent University, Ghent, Belgium; James R. Schmidt, LEAD-CNRS UMR5022, Université de Bourgogne, Dijon, France.
